# Behaviour of fattening pigs fed with liquid feed and dry feed

**DOI:** 10.1186/s40813-015-0009-7

**Published:** 2015-10-13

**Authors:** Mate Zoric, Sven-Erik Johansson, Per Wallgren

**Affiliations:** 1 Department of Animal Health and Antimicrobial Strategies, National Veterinary Institute, SE-751 89 Uppsala, Sweden; 2 Department of Clinical Sciences, Faculty of Veterinary Medicine and Animal Sciences, Swedish University of Agricultural Sciences, SE-750 07 Uppsala, Sweden; 3 Nibble Farming, SE-725 95 Västerås, Sweden

**Keywords:** Pig, Feeding, Performance, Behaviour, Dry feed, Liquid feed, Eating time

## Abstract

**Background:**

Facilities for fattening pigs offer limited possibilities for exploration and wet feeding systems, where the pigs drink the food instead of eating it, have expanded on behalf of dry feeding systems. As little has been made to evaluate liquid feeding from the point of view of the pigs, the aims of this study were to compare behaviour in general and behaviour at feeding in particular of fatteners offered dry or wet feed. The study was carried out in an integrated herd with age segregated rearing of pigs and access to both feeding systems in the fattening units. Apart from the feeding system, the pens were identical and they were managed by the same staff. Pigs were allocated to the fattening units at 20 kg body weight and their behaviour was studied through web cameras during day hours (07.00 to 19.00).

**Results:**

Pigs performed well in both systems, but differed in behaviour. Fattening pigs offered dry feed spent longer time (*P* < 0.001) eating at every feeding occasion. They also expressed fewer regroupings during the first week (*P* < 0.001) when the social rank not yet was established. Pigs fed liquid feed regrouped during the effective eating time, while pigs fed dry feed regrouped when the first pig already had left the through which rather reflected seeking for leftover feed. Restlessness was rarely recorded before feeding, but rather frequently afterwards. During week 5–9 restlessness was more frequently recorded among pigs offered wet feed. Pigs offered liquid feed expressed unwanted behaviour in terms of belly-nosing, and nibbling of ear or tail to a somewhat higher extent than pigs offered dry feed. In both systems the pigs were occupying themselves with straw when offered, but only as long as they regarded it as new, *i.e.,* for around 45 min following each provision.

**Conclusions:**

Pigs performed equivalent in both systems, but from an animal welfare view we recommend dry feed to growing pigs and suggest that liquid feed systems ought to be reserved for systems using alternative feed supplies like whey or other liquid leftovers from the food industry.

## Background

Pigs are omnivorous animals that under natural conditions spend a large part of their active time searching for food [1]. Exploring with a distinct purpose of finding food or an attractive resting place reflects extrinsic explorations. Pigs may also express intrinsic exploratory behaviours, which mean exploring to gather general information on their surroundings [2, 3]. The combination of intrinsic and extrinsic behaviours adapts the pig to its environment and they show a wide range of behaviours to investigate and manipulate the environment [4, 5].

Due to an aim of reducing production costs and to promote feed utilization by minimizing the physical activity of the pigs, the environments for fattening pigs generally neither provide proper stimulation for appetitive behaviour nor for exploratory behaviour [3]. Lack of space and stimulation may increase aggression among fattening pigs [6], and they may redirect their exploratory behaviour from the surroundings to the pen mates [7]. This may in turn lead to physical injures and stress, that if unresolved can lead to chronic stress with negative consequences for immune functions and productivity [8, 9].

Foraging behaviour (rooting and grazing) accounts for a large proportion of the daily activity of pigs kept in a semi-natural enclosure [10], compared to approximately 5 % in modern conventional production [11]. In addition, wet feeding systems, where the pigs drink the food instead of eating it, have expanded on behalf of dry feeding systems. The aims of this study were to assess the behaviour of fatteners in modern rearing facilities, and to compare the behaviour of pigs offered dry feed with that of pigs offered liquid feed.

## Results

### General

The performance of the pigs in the pens studied was in accordance with the mean of the batch they were reared in. No pig in any of the pens studied were treated for any disease, and the level of aggressiveness was neither judged as higher or lower than in the rest of the batch. In both batches pigs were slaughtered as they reached the market weight of around 115 kg. One third of the pigs in each batch was slaughtered on day 93, 100 and 107, respectively, with a mean of 100 days. Pigs performed equal in both systems with a true daily weight gain of the entire batches of 958 g per day for pigs in the dry feeding system and 950 g per day in the liquid feeing system, with a feed conversion of 32.8 and 34.8 MJ per kg growth, respectively (Table 1). The meat percentage at slaughter was 57.6 and 57.0 %, respectively, and the pathological lesions recorded at slaughter were within the normal range of the abattoir.

**Table 1:** Mean performance of the pigs in the two batches studied

		Dry feed	Wet feed
		(*n* = 302)	(*n* = 328)
Total turnover time of batch including washing	(days)	114	118
Mean day when reaching market weight	(days)	100	100
Weight at installation	(kg)	19.3	19.3
Carcass weight at slaughter	(kg)	85.9	85.4
Meat percentage of carcass	(%)	57.6	57.0
Estimated liv weight at slaughter^a^	(kg)	115.1^a^	114.3^a^
Weight gained during rearing		95.8	95.0
True daily weight gain (100 days)^b^	(g per day)	958^b^	950^b^
Economic daily weight gain (114 and 118 days)^c^	(g per day)	840^c^	806^c^
Feed consumed per pig	(MJ)	3595	3892
Feed conversion	(MJ per kg)	32.8	34.8

^a^Mean carcass weight × 1.34

^b^Mean weight gain / mean age when slaughtered

^c^Mean weight gain / total turnover time including washing and empty period prior to next batch

### Activities

As seen in Table 2, pigs in the wet feeding system were more active during the entire fattening period (*P* < 0.001, λ^2^-test). During the first week after installation, activity was observed during 59 % of the time in the wet feeding system compared to 43 % in the dry feeding system (*P* < 0.001, λ^2^-test). With time, the pigs became less active in both systems due to increased resting and sleeping (Table 2). Pigs in the wet feeding system decreased their activity from 59 % of the time during the arrival week to 35 % 6 weeks later (*P* < 0.001, λ^2^-test), and from 35 % week 6 to 23 % week 13 (*P* < 0.001, λ^2^-test). The overall activity of the pigs in the dry feeding system had decreased (*P* < 0.001, λ^2^-test) to 15 % of the time 6 weeks after arrival, and thereafter remained at that level. At the end of the fattening period, activity was recorded during 23 and 15 % of the time in the wet and dry feeding systems, respectively (*P* < 0.001, λ^2^-test).

**Table 2:** Activity and non-activity of pigs during fattening period presented as a percentage of the total time from 7 am to 7 pm for each parameter

	Non-activity		Activity			
	Resting	Sleeping	Eating^a^	Activity	Nosing^b^	Nibbling^c^
	(%)	(%)	(%)	(%)	(%)	(%)
Installation week 1						
Wet feeding	14.2	26.9	**1.6**	**39.4**	**17.8**	**0.1**
Dry feeding	**12.4**	**44.6**	2.5	38.7	0.5	1.3
Installation week 6						
Wet feeding	9.3	55.6	**1.3**	**30.9**	-	**2.9**
Dry feeding	**43.7**	**41.3**	1.9	11.9	-	1.2
Installation week 13						
Wet feeding	10.3	66.8	**0.7**	**21.8**	-	**0.4**
Dry feeding	**29.7**	**55.4**	1.4	13.0	-	0.5
*Mean of 1, 6 & 13*						
Wet feeding	11.3	49.8	**1.2**	**30.7**	**5.9**	**1.1**
Dry feeding	**28.6**	**47.1**	1.9	21.2	0.2	1.0

The table compare a wet feeding system with a dry feeding system during the first, sixth and thirteenth week of the fattening period. Bolded data represent significantly (*P* < 0.001) higher percentage of the merged parameters for activity and non-activity within observation week than the un-bolded data

^a^Total eating time; ^b^Belly-nosing; ^c^Nibbling tail or ear

### Unwanted behaviours

Unwanted behaviours were observed in both systems (Table 2). Nibbling of tail or ear (not biting) at a low and fairly constant incidence was recorded during the entire period. Suckling of pen mates (belly nosing) was observed during first 5 weeks following installation, but practically disappeared after that. During the first week after arrival, this behaviour was recorded during 17.8 % of the time in the wet feeding system, compared to 0.5 % in the dry feeding system (*P* < 0.001, λ^2^-test).

### Behaviour in relation to feeding system

**Fig. 1:**
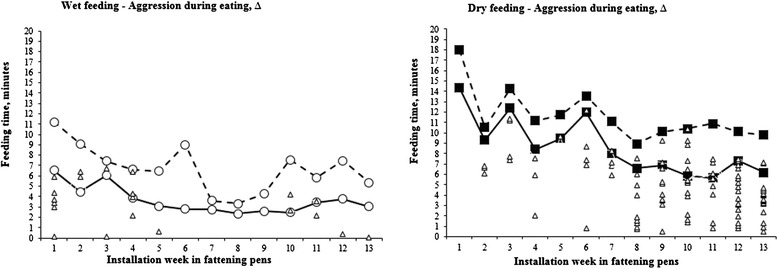
The eating time and aggressions between pigs during the total eating time. Influence of feeding strategy on the effective and total feeding times in minutes during the fattening period. To the left, open symbols represent the wet feeding system while filled symbols represent the dry feeding system to the right. The effective eating time, equal to when the first pig left the feeding through, is shown by full lines. The total eating time is shown by dotted lines, and illustrate when the last pig left the feeding through. Individual aggressions during the total eating time in relation to start of feeding are illustrated by triangles

**Fig. 2:**
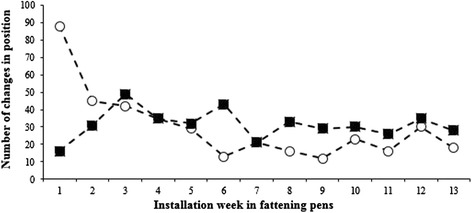
Change of position during the total eating time. The total number of changes in position of pigs during the morning feeding throughout the rearing period. Open symbols represent the wet feeding system and filled symbols represent the dry feeding system. It should be noted that regroupings generally took place earlier after onset of feeding in the wet feeding system than in the dry feeding system. The mean total eating time (*i.e.,* minutes from start until all pigs had left the trough) over the rearing period was 6.7 ± 2.3 min for pigs in the wet foddering system, and 11.6 ± 2.4 min in the dry foddering system

The mean effective eating time per feeding, *i.e.,* when first pig leaved the trough, was 3.6 ± 1.3 min in the wet feeding system compared to 8.6 ± 2.7 min in the dry system (*P* < 0.001, *t*-test) over the rearing period of 13 weeks (Fig. 1). In the wet feeding system, an extensive exchange of positions at the trough was denoted before all pigs had left the trough (Fig. 2). During the total eating time, an exchange of position was on average seen every 15.6 ± 5.5 s in the wet and every 23.5 ± 9.6 s in the dry feeding system, respectively (*P* < 0.001, *t*-test). Figure 1 shows the incidence of aggressions between pigs when eating. These aggressions generally took place early during eating in the wet feeding system, while the number of aggressions during eating increased with time in the dry feeding system.

**Fig. 3:**
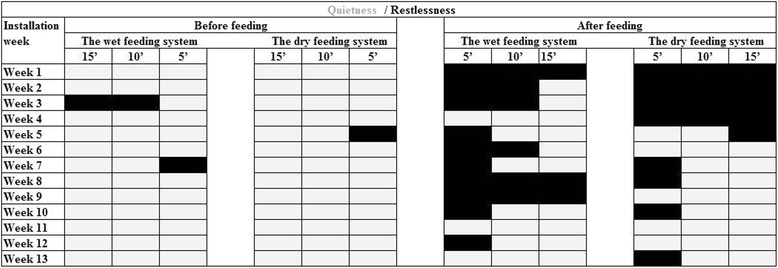
Ambience in pens before and after feeding. The pig pens were registered in periods of five minutes for 15 min before and after feeding. Grey coloured cells represents quietness in the pen and black cells represents restlessness in the pen. During the period from five to 9 weeks after installation, restlessness after eating were more often (*P* < 0.01, λ^2^-test) recorded in pigs fed the liquid diet

Pigs in the both systems remained quiet during the last 15 min prior to feeding throughout the rearing period. In contrast, restlessness post feeding was in both systems recorded during the first three to 4 weeks of the fattening period (Fig. 3). Thereafter restlessness declined (*P* < 0.001, λ^2^-test) in pigs offered dry food, and during week 5 to 9 after installation restlessness post eating was lower (*P* < 0.01, λ^2^-test) in this group. From week 10 and onwards, the restlessness post feeding declined (*P* < 0.001, λ^2^-test) also in pigs offered wet feed.

### Behaviour following provision of straw

Following each provision of straw during the study period of 13 weeks, pigs occupied themselves with it for approximately 45 min (42.6 ± 8.5 min in the dry feeding system and 46.5 ± 9.6 min in the wet feeding system), and thereafter they were uninterested of the straw.

## Discussion

Self-feeding pigs divide their eating and drinking periods randomly throughout the day [11] and since feeding behaviour is stimulated by the sight of other pigs eating group housed pigs consume more feed than individually kept pigs [12]. A*d libitum* fed pigs eat ten to twelve meals per day [13], but fattening pigs are normally fed 2–3 times daily. Further, wet feeding systems have expanded on behalf of dry feeding systems, and today around 70 % of all fattening pigs in Sweden are fed liquid feed [11]. However, little has been made to evaluate liquid feeding from the point of view of the pigs. Therefore, the aims of this study were to compare behaviour in general and behaviour at feeding in particular of fatteners offered dry or wet feed, respectively.

In modern pig production with a time-concentrated feed intake for growers, competition at the feed trough can expose pigs to stress both before, during and also after feeding [14]. In agreement with reports concluding that the eating times correspond to approximately 5 to 10 % of the time that pigs kept in a semi-natural environment spend searching for food [11], the food was rapidly consumed in both systems. However, the eating time in the wet feeding system was around 50 % shorter than in the dry feeding system. Despite this, the number of regroupings during eating was 5.5 times higher during the first week after installation.

Aggressions may be used to establish ranking order within a group [15], but the main cause of aggression between pigs is competition over feed [9, 16] and when the feeding space is limited this is most clearly evident for the smallest pigs [17]. This competition includes fighting and regrouping to get access to feed, and the behaviour of the pigs was influenced by the feeding system. The effective eating time in the wet feeding system, in which most pigs drank the feed instead of chewing it, ranged between two and six minutes. Obviously pigs that were close to the nipples were rewarded with a better access to feed than pigs that had to wait for the liquid feed to spread to the outer parts of the feeding through. As a result a very large number of regroupings with the aim of approaching the nipples were made during the first week. Since the number of regroupings decreased with 50 % the following week, and also the incidence of aggressive interactions that took place as long as the feed lasted ceased with time, the social ranking probably affected the behaviour during eating. Despite this, the regroupings still corresponded to around five regroupings per pig during the short eating time.

In the dry feeding system, where pigs had to chew the feed, the effective eating time was longer, ranging from 7 to 14 min. As the feed was distributed instantly to the entire feeding through there was no reward of a specific eating position, and thereby the regroupings during the effective eating time were fewer in this system. Still, the total number of regroupings were equal or higher in this system from the third week after installation and onwards. However, they took place later during the eating process, or when the first pig already had left the through and rather reflected seeking for leftover feed than active search/fight for feed. Also the number of aggressions increased with rearing time in this system, possibly due to an increased crowding at the feeding through as the pigs increased their body size combined with an understanding that their “own” individual ration was safely located in the feeding through in front of them. In the wet feeding system the drain of the feed from that spot probably prevented aggressive interactions head to head in favour of regrouping attempts.

Restlessness before and/or after eating may indicate frustration or confusion [18]. In both feeding systems, restlessness was only rarely recorded prior to feeding, but rather frequently afterwards. During the first 4 weeks, restlessness post feeding was evident in both groups, but from the 5th to the 9th week restlessness was more frequently recorded in pigs offered the wet feed, peaking at week 8 and 9, just before the feed ration per kg body weight was decreased with the aim of achieving a high meat percentage at slaughter [19, 20]. Possibly low ranked pigs were bigger losers in the wet feed system due to the quicker feed consumption, which in turn could have induced conflicts. In the dry feeding system, pigs were eating during a longer time and the incidence of regroupings during the effective eating was lower. Despite the increased incidences of aggressive interactions during the total eating time with aging in the dry feeding system there was quietness among these pigs after eating from the fifth week and onwards.

Despite that both groups performed well, the feed utilisation was somewhat lower in the wet feeding system. This may have been induced by physiological processes initiated by the higher competition for feed in this system, but it could also mirror an increased waste of feed in the wet feeding system – which in turn also could be dependent on the competition for feed in this system.

Fattening pig environments generally neither provide proper stimulation for appetitive behaviour related to eating, nor for exploratory behaviour in general [3]. Still, the high activity level of the pigs during the first week after transfer to a fattening unit was not unexpected since the pigs explored their new environment, and also established a social hierarchy in the pen during that time [21, 22]. A suckling behaviour (belly-nosing) was seen in both systems during the first 5 weeks, again at a higher degree in the wet feeding system. Interestingly, the age of the pigs when this behaviour disappeared corresponded to the age (17 weeks) when free living pigs are completely rejected by the dam and thereby finally weaned [23]. Apparently belly-nosing, that also has been recorded by others [24, 25], mimic a physiological need of suckling until that age. The incidence of belly-nosing appears not to be influenced by any diet whatsoever [24], but may be reduced by nose enrichments [25], which may explain the difference between the two feeding systems. Apparently the motivation to suckle will remain high when piglets are removed from the sow before their natural weaning age, especially when placed in a stimulus-deprived environment [25].

If there is nothing to explore in a pig pen, the pigs may redirect their exploratory behaviour towards pen fixtures or pen mates, and in worst case develop stereotypic manners [1]. No stereotypic behaviour was however recorded in this study. The incidence of unwanted behaviours with a potential to evolve into stereotypic behaviour (nibbling of tail or ear) were restricted to minutes of the day throughout the study, and appeared as a form of exploration or game throughout the rearing period - although at a higher level among pigs in the wet feeding system at the middle of the rearing period. Another effect of low-stimuli environments may be inactive or apathetic pigs. Indeed, the activity of the pigs in the present study decreased with aging in both feeding systems. However, it should also be remembered that the activity of pigs generally is considered to decrease with age [26–28].

In both systems the pigs spent around 45 min occupying themselves with the straw after each provision. However, after that time they lost interest to the straw, and the conclusion was that the pigs spent interest to the straw just as long as they considered it to be new and unsullied as also concluded by others [29]. Thus a way to stimulate the exploratory behaviour of fatteners could be to more frequently offer small amounts of straw, and/or to let them actively work for feed through automatics [30]. Another way to keep fatteners busy could of course be to offer them feed more often, but considering the short eating times recorded, the value of that could be discussed.

## Conclusions

Modern fattening facilities offer limited possibilities for pigs to express natural behaviour, and the consumption of feed is intense. Pigs performed equal in the two systems, but pigs offered dry feed spent longer time eating at every feeding occasion than pigs offered liquid feed. They also expressed fewer regroupings during feeding and expressed unwanted behaviour in terms of belly-nosing, restlessness after feeding and nibbling of ear or tail to a somewhat lower extent than pigs offered liquid feed. From an animal welfare view we therefore recommend dry feed to growing pigs, and suggest that liquid feed systems ought to be reserved for systems using alternative feed supplies like whey or other liquid leftovers from the feed industry. Further, the transient interest to straw indicates a desire of change among pigs, which ought to be taken into account when designing new rearing systems.

## Methods

### Animals and housing

The study has been approved by the Ethical Committee for Animal Experiments, Uppsala, Sweden (reference number C 147/2010; Patient-related studies / studies in animals. Scientific work performed in connection with normal veterinary disease investigation and similar situations). The study was performed in a conventional farrow to finish farm with 280 Swedish Yorkshire x Swedish Landrace sows mated with Duroc semen. Piglets born were weaned at 32 days of age and had free access to a home produced dry creep feed with 15 % protein from ten days of age until allocation. The farm used an all-in/all-out production system from birth to slaughter. The daily weight gain and feed conversion were recorded at batch level, and each unit was cleaned and left empty around 1 week before restocking.

Pigs were allocated to the fattening units at a weight of around 20 kg. Each pen housed nine pigs. The pens measured 3.0 × 1.8 m with a concrete floor, and an additional dunging area of 1.5 × 1.2 m with slatted floor, giving a total area of 0.8 m^2^ per pig [31]. The length of the feeding trough was 3.0 m, giving enough feeding space for all pigs to simultaneously get access the trough. The herd had no specific health problems and the number of pathological lesions registered at slaughter was within the normal national range.

**Fig. 4:**
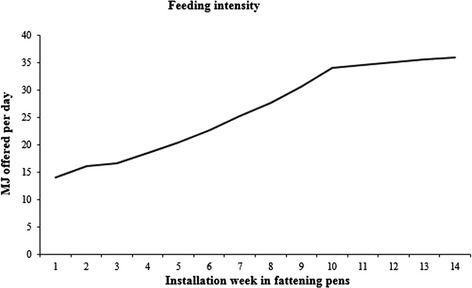
Feeding intensity. MJ offered per pig and day throughout the rearing period in both batches. The feed was identical in both feeding systems except that the dry feed was diluted with water in the liquid feeding system

There were two feeding systems for fatteners in the herd. A dry feeding system was applied in two units with 36 pens each and a wet feeding system was applied in two units with 42 pens each. In both systems, the pigs were fed twice a day according to the norm recommended by the Swedish University of Agricultural Sciences, the so called SLU-norm [32]. The pigs were fed 14.0 MJ per pig and day when transferred to the fattening pens at 19.3 kg body weight. There was a gradual increase up to 34.1 MJ per day after 10 weeks, and thereafter the ration offered remained at that level (Fig. 4). The feed was identical in the both systems except that it was diluted with water in the liquid feeding system. A concentrate (Slaktex, Lantmännen, Svalöv, Sweden) was mixed with home produced cereals to qualities described in Table 3. The mineral and vitamin dosages were adjusted according to the SLU-norm [32]. Approximately 2 kg of chopped straw was scattered on the floor per pen once daily after cleaning the pens.

**Table 3:** Energy, protein and dry matter content of the feed. The wet feed was dry feed diluted with water as described in the table

		Dry feed	Wet feed
*Phase I; up to 70 kg body weight*			
Dry matter content	(%)	88.6	28.1^a^
Energy content	(MJ per kg)	12.61	4.07^a^
Protein content	(%)	17.3	5.59^a^
*Phase II; from 70 kg body weight*			
Dry matter content	(%)	88.6	27.4^b^
Energy content	(MJ per kg)	12.69	4.35^b^
Protein content	(%)	15.6	5.35^b^

^a^31.72 % dry feed diluted with 68.28 % water; ^b^30.93 % dry feed diluted with 69.07 % water

### Study design, behavioural observations and statistical analysis

Pigs in the two feeding systems (liquid and dry) had identical pens, including water nipples, and were handled by the same staff. One pen with nine pigs in each system was videotaped at Tuesdays from arrival at a weight of 19.3 kg weight until the first pigs reached market weight 13 weeks thereafter. One pen in the middle row of each unit studied was selected by random and filmed during the daylight hours, from 7 am to 7 pm. Pigs are normally more active during daylight hours [33], and therefore it seemed reasonable to study behaviours during that time. The web camera photographed one picture every second, corresponding to 43,200 photos per day and pen studied. For each day studied, the behaviour of the pigs in the pens studied was registered and summarized either as minutes or as percentages of the 12 h studied.

The behaviour of the pigs was defined as one out of six previously defined manners, and the behaviour of the majority of the pigs was recorded at each observation. The six defined manners were:

1. Activity
  (standing, walking, drinking, rooting);
2. Resting
  (lying without sleeping; Pigs stay at one place but may be roused easily by environmental changes);
3. Sleeping
  (posture lying and eyes closed. Pigs are not alert to environmental changes);
4. Eating
  (the effective eating time was defined as the time from onset of feeding until the first pig left the though; and the total eating time was defined as the time from onset of feeding until the last pig left the through);
5. Nibbling tail or ear of a pen mate;
6. Belly-nosing, *i.e.,* suckling of pen mates.

Regroupings and aggressions were recorded independently from the six behaviours described above. As each regrouping only included one pig that regrouped for a better position (although other pigs could be affected by them), and each aggression only included two pigs (aggressor and receiver), the recording system based on the behaviour of the majority of the pigs did not suite these parameters. As these behaviours in principle only were recorded during feeding (data not shown), regroupings (changing position) and aggressions (active confronting of another pig) during the total eating time were recorded as number per feeding.

Further, the activities of the pigs were registered in periods of five minutes for 15 min before and after feeding. During these periods, the ambience in the pens was validated using an ethogram including sleep, rest, activity, regrouping and aggressions as parameters. The outcome of the ethogram was defined as quietness or restlessness for each period, where quietness was noted when sleep, lying/or a peaceful standing dominated whereas restlessness was recorded when turbulent standing, regroupings and aggressions dominated.

Finally, the behaviour following provision of straw in terms of pigs occupying themselves with the straw was recorded in detail every day studied. The total time when the pigs were interested in the straw was defined as the time from onset of provision of straw until the majority of the pigs had left the straw.

As a control, incidences of diseases and aggressiveness as well as visible growth performance of the entire batches scrutinised were recorded, and the performance of the pigs in the pens studied were compared with that of the total batch.

Statistical calculations carried out were *t*-tests and chi-square tests, using the Stata Program (Stata/IC 11.2 Statistics/Data Analysis, StataCorp LP, 4905 Lakeway Drive College Station, Texas 77845, USA). All statistical analyse performed were based on observations with complete recordings.

The study has been approved by the Ethical Committee for Animal Experiments, Uppsala, Sweden (reference number C 147/2010; Patient-related studies/studies in animals. Scientific work performed in connection with normal veterinary disease investigation and similar situations). The study included filming of pigs without any alterations in their environment, with the aim to study the behaviour of growing pigs. In addition, data concerning performance and disease registrations at slaughter were collected.
